# IL-6 signaling contributes to cisplatin resistance in non-small cell lung cancer via the up-regulation of anti-apoptotic and dna repair associated molecules

**DOI:** 10.18632/oncotarget.4753

**Published:** 2015-07-30

**Authors:** Shanzhou Duan, Ying Tsai, Peter Keng, Yongbing Chen, Soo Ok Lee, Yuhchyau Chen

**Affiliations:** ^1^ Department of Radiation Oncology, University of Rochester School of Medicine and Dentistry, Rochester, NY 14642, USA; ^2^ Department of Cardiothoracic Surgery, The Second Affiliated Hospital of Soochow University, Suzhou, Jiangsu 215004, P.R. China

**Keywords:** non-small cell lung cancer, IL-6, cisplatin resistance, apoptosis, DNA repair

## Abstract

Cisplatin-based chemotherapy is currently the most effective treatment regimen for non-small cell lung cancer (NSCLC), but eventually tumor resistance develops which limits its success. The potential implication of IL-6 signaling in the cisplatin resistance of NSCLC was explored by testing whether NSCLC cells with different levels of intracellular IL-6 show different responses to the cytotoxic treatment of cisplatin. When the cisplatin cytotoxicity of the IL-6 knocked down human NSCLC cells (A549IL-6si and H157IL-6si) were compared with their corresponding scramble control cells (A549sc and H157sc), higher cisplatin cytotoxicity was found in IL-6 si cells than sc cells. Subcutaneous xenograft mouse models were developed using a pair of A549sc and A549IL-6si cells. When the tumor grew to about 400 mm^2^, mice were treated with cisplatin and tumor regression was monitored. Higher tumor regression was detected in the A549IL-6si xenografts compared to A549sc xenografts following cisplatin treatment. Immunostaining study results from tumor tissues also supported this finding. Expression of anti-apoptotic proteins Bcl-2 and Mcl-1 and DNA repair associated molecules ATM, CHK1, TP73, p53, and ERCC1 were significantly up regulated in cisplatin-treated A549sc and H157sc cells, but no increase was detected in A549IL-6si and H157IL-6si cells. Further inhibitor studies revealed that up regulation of these molecules by IL-6 may be through activation of IL-6 downstream signaling pathways like Akt, MAPK, Stat3, and Erk. These results provide potential for combining cisplatin and inhibitors of IL-6 signaling or its downstream signaling pathway as a future therapeutic approach in preventing development of cisplatin resistant NSCLC tumors.

## INTRODUCTION

Lung cancer is the most common cause of cancer death in both men and women [1]. It is heterogeneous and histologically divided into two types: small cell lung carcinomas (SCLCs) and non-small cell lung carcinomas (NSCLCs), with the latter comprising 85% of lung cancer cases [2]. NSCLCs also constitute a heterogeneous population of tumors, including squamous, adenocarcinoma, and large cell carcinomas [1]. Despite recent progress, the therapeutic outcome of lung cancer remains poor.

Platinum-based drugs, particularly cis-diammine-dichloroplatinum (II) (cisplatin, DDP), are used in the treatment of many cancers, including lung cancer. Initially, cisplatin treatment demonstrates favorable outcomes, but often chemoresistance develops later on and results in failure of this therapy.

The development of cisplatin resistance is mediated through multiple mechanisms. It has been well reviewed in the literature that DNA lesions and the induction of mitochondrial apoptosis are the most critical mechanisms of cisplatin action [3, 4]. In addition, enhancement of DNA repair activity has been observed in cisplatin resistant cell lines [5]. Involvement of DNA repair defects in the treatment of NSCLC by cisplatin has also been reported [6]. Recently it was also reported that hyperactivation of PARP was involved in cisplatin resistance in lung cancer cells [7] and inhibition of PARP could selectively increase the cellular sensitivity to cisplatin [8]. Meanwhile, the use of genetic variation in predicting the overall survival of lung cancer patients receiving platinum-based chemotherapy has been reported [9]. Cisplatin resistance can also be triggered by altered drug delivery system, metabolism, and tumor microenvironmental changes such as hypoxia [10].

IL-6 (Interleukin-6) is a cytokine that produces a broad range of cellular and physiological responses upon activation, and has been implicated in the tumorigenesis of epithelial cancer [11]. The implication of IL-6 in NSCLC progression has previously been suggested. In a study by Yanagawa *et al* [15], IL-6 was detectable in 29 patients with lung cancer (39%), but not in any of the patients with benign lung diseases. Yamaji *et al* [16] investigated the correlation between IL-6 production and tumor proliferation in NSCLC, and found 53% of NSCLC cell lines express IL-6 mRNA and protein. The circulating IL-6 level has also been suggested as a prognostic marker for survival in advanced NSCLC patients treated with chemotherapy [17].

In this study, we investigated whether IL-6 signaling is important in mediating cisplatin-resistance in NSCLC. We also revealed the molecular mechanisms by which IL-6 contributes to this cisplatin-resistance.

## RESULTS

### Increased cisplatin cytotoxicity in IL-6 knocked down (si) A549 and H157 cells compared to scrambled control (sc) cells

**Figure 1:**
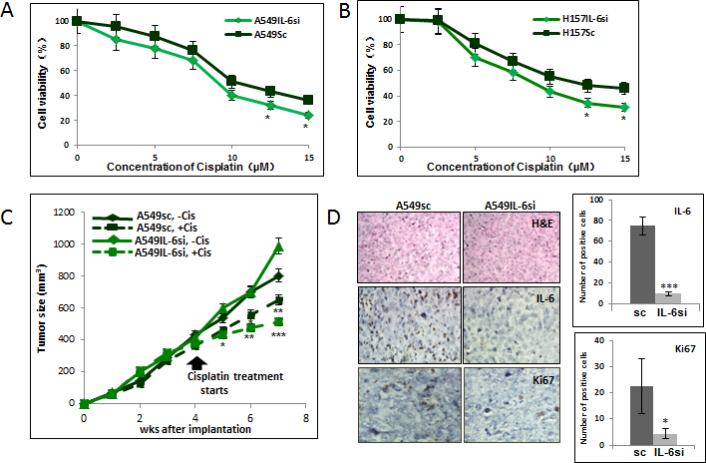
IL-6 knockdown in NSCLC cells increased *in vitro* and *in vivo* sensitivity to cisplatin **A.** Cisplatin cytotoxicity tests of A549IL-6si/sc cells upon various concentrations of cisplatin for 2 days. **B.** Cisplatin cytotoxicity tests of H157IL-6si/sc cells upon various concentrations of cisplatin for 2 days. **C.** Tumor regression analyses of A549IL-6si and A549sc cells-derived xenografts in nude mice on cisplatin treatments. Xenografts were developed by subcutaneously injecting 1 × 10^6^ A549IL-6si or A549sc cells into flanks of 8 week old female nude mice. When tumor volumes reached 400 mm^3^, cisplatin (3 mg/kg, i.p. two times per week) treatment started. Tumor growth was monitored twice per week and at the end of three weeks of treatment, mice were sacrificed. **D.** H&E and IHC staining of tumor tissues. Tumor tissues of A549IL-6si/sc xenografts were processed and subjected to H&E and IHC staining. Upper panels show H&E staining, middle panels present IHC staining with IL-6 antibody, and lower panels are the IHC staining results using antibody against Ki67 (magnification, 100x). Quantitation of IL-6 and Ki67 IHC staining is shown on right. *p < 0.05, **p < 0.01, ***p < 0.001.

To investigate the effects of intracellular levels of IL-6 on the sensitivity to cisplatin cytotoxicity in NSCLC cells, we manipulated the IL-6 levels of A549 and H157 NSCLC cell lines *in vitro* by lentiviral transduction. We obtained the IL-6 knocked down cells (A549IL-6si and H157IL-6si) together with their respective scrambled control cell lines (A549sc and H157sc). High knockdown efficiency (more than 90%) was confirmed in both IL-6si cell lines by qPCR and IL-6 ELISA analyses (data not shown). When cisplatin cytotoxicity was tested in these cells using the MTT assay, it was found that A549 and H157 cells with knocked down IL-6 were more sensitive than their scrambled control cells (Fig. 1A, 1B), suggesting that NSCLC cells showed different responses to cisplatin treatment depending on their intracellular IL-6 level and reduced level of intracellular IL-6 made cells more sensitive to cisplatin.

### Tumors derived from IL-6 knocked down cell showed more growth regression than those derived from IL-6 expressing cells following cisplatin treatment

To confirm the *in vitro* results showing higher cisplatin cytotoxicity in IL-6 knocked down cells, we performed xenograft studies by implanting A549IL-6si and A549sc cells subcutaneously into nude mice. When tumors reached the size of 400 mm^3^, we injected mice with cisplatin (3 mg/kg, i.p.) twice a week for 3 weeks and monitored the subsequent tumor growth. A significant reduction in tumor growth was detected in A549IL-6si cell-derived xenografts than those from A549sc cells following cisplatin injection (Fig. 1C). These results suggest that the IL-6 knockdown makes tumor cells more sensitive to cisplatin, and are consistent with the *in vitro* cytotoxicity results (Fig. 1A).

We further analyzed the expression of several molecules in tumor tissues to determine the relationship between IL-6 and cisplatin sensitivity. Fig. 1D shows the H&E staining in the upper panel and the IL-6 immunohistochemical (IHC) staining in the middle panel, confirming the IL-6 knockdown status in A549IL-6si cell-derived tumors. When the tumor tissues were stained with the antibody of the proliferation marker Ki67, a lower number of Ki67 positively-stained cells were detected in A549IL-6si cell-derived tumors (Fig. 1D, lower panel), which is consistent with the tumor growth data (quantification of IL-6 and Ki67 staining results are shown to the right of Fig. 1D).

### IL-6 protected NSCLC cells from cisplatin-induced apoptosis via up-regulation of anti-apoptotic proteins

**Figure 2:**
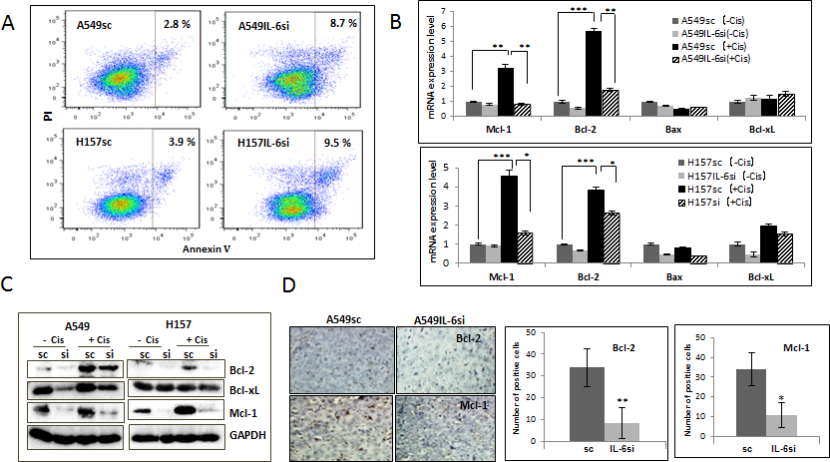
IL-6 protected A549 and H157 cells from apoptotic death after cisplatin treatment via up regulation of Bcl-2 and Mcl-1 **A.** Analysis of apoptosis following cisplatin treatment. A549IL-6si/sc and H157IL-6si/sc cells were treated with cisplatin (5 μM) for 48 hours and subjected into Annexin V based-flow cytometric analysis according to the manufacturer's instruction. The % of apoptotic cell is indicated in each graph. **B.** qPCR results of analyzing mRNA expressions of anti-apoptotic molecules in A549IL-6si/sc and H157IL-6si/sc cells after cisplatin treatment (5 μM, 48 hours). **C.** Western blot results showing expression of anti-apoptotic proteins in A549IL-6si/sc and H157IL-6si/sc cells following cisplatin treatment (5 μM, 48 hours). **D.** IHC staining of tumor tissues of A549IL-6si/sc xenografts showing expression of Bcl-2 and Mcl-1(Magnification, 100x). Quantitation of Bcl-2 and Mcl-1 IHC staining is shown on right. *p < 0.05, **p < 0.01, ***p < 0.001.

One of the molecular mechanisms of cisplatin resistance is the blocking of cisplatin-induced apoptosis by IL-6 [3]. To investigate whether the level of IL-6 expression affects the apoptotic cell death in NSCLC cells following cisplatin treatment, A549IL-6si/sc and H157IL-6si/sc cells were treated with cisplatin for 48 hours and the apoptotic cell death was analyzed using the AnnexinV-based flow cytometric method. As shown in Fig. 2A, more apoptotic death was detected in IL-6si than sc cells (8.7% vs. 2.8% for A549 cells and 9.5% vs. 3.9% for H157 cells, respectively).

We then investigated whether the difference in apoptotic death produced by cisplatin was due to the IL-6 effect on expression of pro- and anti-apoptotic molecules. When the expression of mRNAs (Fig. 2B) and proteins (Fig. 2C) of the pro-apoptotic (Bax) and anti-apoptotic (Bcl-2, Bcl-xL, and Mcl-1) molecules were measured in the IL-6si/sc cell pairs, we found a significant increase in Bcl-2 and Mcl-1 expression (not Bcl-xL and Bax) in cisplatin-treated sc cells but not in si cells. These results suggest that intracellular IL-6 is involved in mitigating damage produced by cisplatin and is responsible for the cellular resistance to it. This protection effect may be attributed through the up-regulation of Bcl-2 and Mcl-1(anti-apoptotic) molecules. The IHC staining of tumor tissues obtained from cisplatin-treated A549 xenografts supported the *in vitro* results by showing a higher number of Bcl-2 and Mcl-1 stained cells in tumors derived from sc cells than those from IL-6si cells (Fig. 2D, quantification is shown on the right).

### IL-6 protected NSCLC cells from cisplatin-mediated DNA damage via up-regulation of DNA damage repair associated molecules

**Figure 3:**
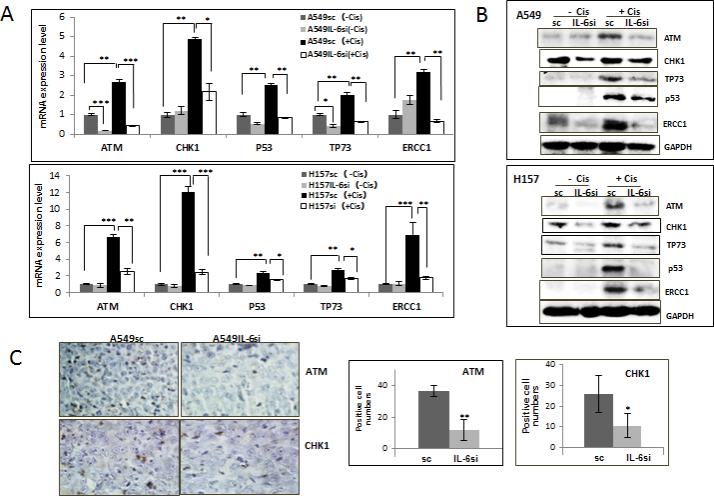
IL-6 expressing A549sc and H157sc cells showed higher expression of DNA repair molecules upon cisplatin treatment compared to A549IL-6si and H157IL-6si cells **A.** qPCR results of mRNA expressions of DNA repair associated molecules in A549IL-6si/sc and H157IL-6si/sc cells after cisplatin treatment (5 μM, 48 hours). **B.** Western blot analyses results showing expression of the DNA repair-associated molecules in A549IL-6si/sc and H157IL-6si/sc cells on cisplatin treatment (5 μM, 48 hours). **C.** IHC staining of tumor tissues of A549IL-6si/sc xenografts investigating expression of ATM and CHK1 (Magnification, 100x). Quantitation of positively stained cells is shown on right. *p < 0.05 **p < 0.01, ***p < 0.001.

The effect of IL-6 on DNA repair molecules of cisplatin-treated cells was investigated to determine if IL-6 induced cisplatin resistance is through the repair of cisplatin-induced DNA damage. It has been shown that cellular responses to DNA damage are coordinated initially through the signaling cascades, the Ataxia telangiectasia mutated (ATM)-CHK [3]. Therefore, the expression levels of the ATM and CHK1 molecules were first tested in A549IL-6si/sc and H157IL-6si/sc cells after they were treated with 5 μM cisplatin for 48 hours. As shown in Fig. 3A (mRNA level) and Fig. 3B (protein level), expression levels of ATM and CHK1 were significantly up-regulated in the IL-6 expressing A549sc and H157sc cells following cisplatin treatment, but not in A549IL-6si and H157IL-6si cells. In addition, the expression levels of p53 and p73, which are well-known downstream targets of ATM that can lead to the apoptosis [23, 24], and the DNA excision repair protein ERCC1 [25–27] were also investigated in this study. Similarly to the results of ATM and CHK1, expression of all these molecules were significantly up-regulated in the IL-6 expressing A549sc and H157sc cells following cisplatin treatment, but not in A549IL-6si and H157IL-6si cells (Fig. 3A and 3B). All these results suggest that IL-6 signaling is important in the repair of the cisplatin-induced DNA damage and contributes to higher survival rates. IHC staining data obtained from tumor tissues also supported our hypothesis by showing a higher number of ATM and CHK1 stained cells in the A549sc xenografts than in the A549IL-6si xenografts (Fig. 3C).

### The IL-6 mediated up regulation of the anti-apoptotic and DNA repair associated molecules may be through IL-6 downstream signaling pathways, such as MAPK/Stat3/Akt/Erk

**Figure 4:**
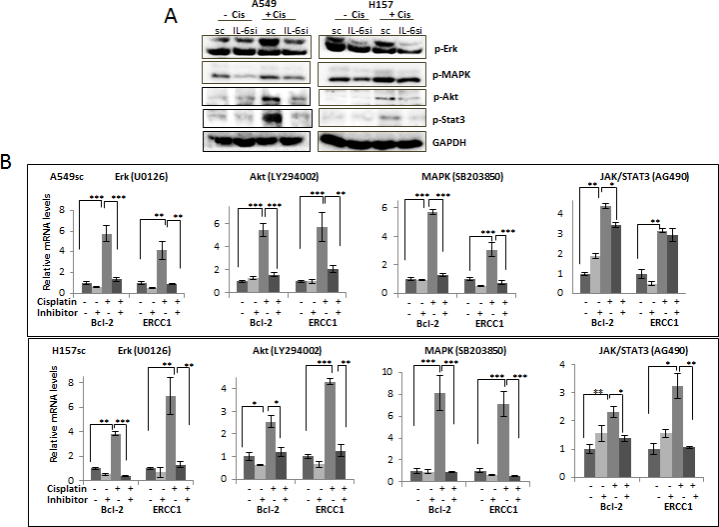
Activation of IL-6 downstream signaling pathways on cisplatin treatment **A.** Western blot analysis analyzing activations of several signaling pathways in A549IL-6si/sc and H157IL-6si/sc cells 48 hours after cisplatin treatments (5 μM). **B.** Analyses of qPCR results showing expression of Bcl-2 and ERCC1 in A549sc and H157sc cells, with or without cisplatin treatment, in the absence and presence of inhibitors of each signaling pathway. *p < 0.05 **p < 0.01, ***p < 0.001.

It has been reported that IL-6 can activate signaling pathways of Stat3, Erk, MAPK, and Akt [12] [28] [29]. When we investigated activation of these pathways, with or without cisplatin treatment, we found they were all highly activated in IL-6 expressing A549sc and H157sc cells upon cisplatin treatment (5 μM, 72 hours), but not significantly elevated in the IL-6 knocked down A549IL-6si and H157IL-6si cells (Fig. 4A).

We then investigated whether IL-6 mediated up regulation of the anti-apoptotic and DNA repair-associated molecules by cisplatin are through the activation of these signaling pathways. Inhibitors of each signaling pathway (SB203850, LY294002, AG490, and U0126 for inhibiting the MAPK, Akt, JAK/Stat3, and MEK/Erk pathway, respectively) were added to the IL-6 expressing A549sc and H157sc cells, and then to test whether the up regulation of two selected molecules, Bcl-2 and ERCC1, by cisplatin can be blocked by the inhibitors. As shown in Fig. 4B, we found that adding inhibitors of the MAPK, Akt, and MEK/Erk pathways almost completely blocked the up regulation of Bcl-2 and ERCC1 molecules, while a lesser effect was observed when the inhibitor of the JAK/Stat3 pathway was used. From these results, we conclude that the activation of Stat3, Erk, MAPK, and Akt signaling pathways are involved in triggering the IL-6 effect on the up regulation of the anti-apoptotic and DNA repair-related molecules, but that the MAPK, Akt, and MEK/Erk signaling pathways are more critical than the JAK/Stat3 pathway.

## DISCUSSION

Development of cisplatin resistance leads lung cancer into a more advanced stage. Oliver *et al* [30] showed advanced histopathology and increased frequency of genomic alterations in the cisplatin resistant lung tumors. Hsieh *et al* [31] also showed acquisition of an enhanced aggressive phenotype in cisplatin resistant NSCLC cells. In addition, Mitsumoto *et al* [32] demonstrated significantly enhanced metastatic properties from *in vivo*-established cisplatin resistant tumors, and Barr *et al* [33] showed that cisplatin resistant NSCLC cell lines displayed a stem cell-like signature which may contribute to lung cancer progression. Therefore, deterring the development of cisplatin-resistance is an urgent issue in clinical settings where cisplatin is the preferred therapeutic option.

We showed in a series of *in vitro* and *in vivo* mice studies that NSCLC cells have varied sensitivity to cisplatin depending on the level of intracellular IL-6. We observed that IL-6 signaling up-regulated expression of anti-apoptotic (Bcl-2, Mcl-1) and DNA repair-associated molecules (ATM, CHK, ERCC1, p53, TP73) upon cisplatin treatment while had little effects on the IL-6 knocked down cells. This finding led us to believe that whether lung cancer cells respond to cisplatin treatment and trigger their survival mechanisms depend on the IL-6 expression levels in the cells. Therefore, the IL-6 expressing NSCLC cells may acquire cisplatin resistance and have a higher chance to survive than cells with a lower level of or no intracellular IL-6.

We also found from the series of inhibitor studies that the up regulation of the genes related to the anti-apoptotic and DNA repair processes by IL-6 is through activation of signaling pathways, including Stat3, Akt, MAPK, and Erk. The involvement MEK/ERK signaling [34], p38 MAPK [35], EGFR-Stat3 signaling [36], and Akt signaling [37, 38], in modulating the DNA repair process has been previously suggested. Nonetheless, we cannot exclude the possibility of direct IL-6 modulation of these molecules at the transcriptional level or through interaction with other molecules, so further studies are necessary to confirm this.

In addition to the altered apoptotic process and modulation of the DNA repair system, there must be other ways that IL-6 modulates sensitivity/resistance to cisplatin, such as altered drug uptake, drug delivery system and metabolism, or tumor microenvironment changes [30, 39]. Whether IL-6 signaling can trigger these mechanisms needs to be elucidated in the future.

In our studies, we found that the intracellular level of IL-6 is critical in protecting cells from apoptosis and promoting DNA repair upon cisplatin treatment. Our results suggest that IL-6 autocrine signaling is important. Since half of the NSCLC cells were reported to secrete IL-6 endogenously [16] and considering heterogeneity of tumors consisting mixture of IL-6-expressing and non-IL-6-expressing cells, the IL-6 autocrine role seems important. However, IL-6 can also be secreted from several cell types comprising the tumor microenvironment such as macrophages [40], endothelial cells [41], and cancer fibroblasts [42], so the paracrine role of IL-6 [43] will also be important. Revealing the paracrine IL-6 role in triggering cisplatin-resistance is a future project.

We suggest that targeting IL-6 signaling inhibits or delays cisplatin-resistance in cisplatin-based chemotherapy. An IL-6 (or downstream signaling) targeting strategy combined with conventional cisplatin therapy may reduce or inhibit cisplatin resistance. Further animal experiments need to be performed to test this therapy outcome.

There have been several attempts to overcome cisplatin resistance in NSCLC cells. Wu *et al* [44] suggested using an inhibitor of mTOR signaling to overcome cisplatin-resistance. Heavey *et al* [45] reported targeting the PI3K-NFκB pathway for battling cisplatin resistance. In addition, Andriani *et al* [46] showed increased sensitivity to cisplatin after applying a FHIT gene into the NSCLC cells, and Bian *et al* [47] reported increasing cisplatin sensitivity of NSCLC cells by up-regulation of miRNA451. Whether IL-6 targeting therapy will overcome acquired cisplatin resistance will be a future project.

## MATERIALS AND METHODS

### Cell culture

A549 and H157 cell lines were purchased from the American Type Culture Collection (ATCC, Manassas, VA) and cultured in RPMI 1640 with 10% FBS. All cells were maintained in a humidified 5% CO_2_ environment at 37°C. For inhibition studies, LY294002 (10 μM) (Sigma, St Louis, MO), SB203850 (10 μM) (Sigma, St Louis, MO), AG490 (5 μM) (Cell Signaling, Danvers, MA), and U0126 (10 μM)(Cell Signaling, Danvers, MA) that inhibit the Akt, MAPK, JAK/Stat3, and MEK/Erk pathways, respectively, were added into the culture before cisplatin treatment.

### Development of IL-6 knocked down and sc control cells by lentiviral transduction

For incorporation of IL-6 siRNA or sc plasmids into A549 and H157 cells, lentivirus construct carrying either sc or IL-6 siRNA (pLenti-II vector, Applied Biological Materials Inc, Canada) was transfected into 293T cells with a mixture of pLent-II-IL-6 siRNA, psPAX2 (virus-packaging plasmid), and pMD_2_G (envelope plasmid) (4:3:2 ratio) using PolyFect Transfection reagent (Qiagen, Valencia, CA). After A549 and H157 cells were virally infected overnight, the culture media containing the virus was replaced with normal culture media, and maintained under normal cell culture conditions. After sub culturing cells, the IL-6 knocked down cells were selected by culturing cells in the presence of puromycin (2 μg/ml) (Sigma) and then maintained in media containing 0.1 μg/ml puromycin.

### Cisplatin cytotoxicity test

Cisplatin cytotoxicity was analyzed by MTT (3-[4, 5-dimethylthiazol-2-yl]-2, 5-diphenyltetrazolium bromide, 5 mg/ml, Sigma, USA) assay. Cells (A549IL-6si/sc and H157IL-6si/sc) were seeded on 96-well plates (7 × 10^3^ cells/well) and treated with various concentrations of cisplatin for 48 hours. MTT test was then performed and absorbance at 490 nm was measured. Cell viability was calculated using the formula: OD sample/OD blank control × 100. Triplicate experiments were performed and average values with mean ± SEM were represented.

### Flow cytometric analysis of Apoptosis

Cells (A549IL-6si/sc and H157IL-6si/sc) were seeded on 24-well plates (2 × 10^4^ cells/well), treated with various concentrations of cisplatin for 48 hours, and their apoptotic death rates were analyzed by the AnnexinV based apoptosis kit (eBioscience, San Diego, CA) according to the manufacturer's instructions.

### RNA extraction and quantitativereal-time PCR (qPCR) analysis

Total RNA (1 μg) was subjected to reverse transcription using Superscript III transcriptase (Invitrogen). qPCR was conducted using the appropriate primers (sequence is provided in Table 1) and a Bio-Rad CFX96 system with SYBR green to determine the mRNA expression levels of genes of interest. Expression levels were normalized to GAPDH level.

**Table 1:** Primer sequences of molecules used in qPCR analyses

Primer name	Sequence (5′ - > 3′)
IL-6 forward	AAGCCAGAGCTGTGCAGATGAGTA
IL-6 reverse	TGTCCTGCAGCCACTGGTTC
Bcl-2 forward	GGTGGACAACATCGCCCTGTGG
Bcl-2 reverse	CAGAGTGATGCAGGCCCCGAC
Bax forward	GGTTATCTCTTGGGCTCACAAG
Bax reverse	TGATGGACGGGTCCGGGGAGCA
Bcl-xl forward	TTGGACAATGGACTGGTTGA
Bcl-xl reverse	GTAGAGTGGATGGTCAGTG
Mcl-1 forward	GGAGGAGGACGAGTTGTAC
Mcl-1 reverse	AAGGCACCAAAAGAAATG
ATM forward	CAGGGTAGTTTAGTTGAGGTTGACAG
ATM reverse	CTATACTGGTGGTCAGTGCCAAAGT
CHK1 forward	CTGGGATTTGGTGCAAACTT
CHK1 reverse	GCCCGCTTCATGTCTACAAT
P53 forward	GGAGGTTGGCTCTGACTGTACC
P53 reverse	TCCGTCCCAGTAGATTACCAC
TP73 forward	GCACCACGTTTGAGCACCTCTGG
TP73 reverse	AGATGTAGTCATGCCCTCCAGGTG
ERCC1 forward	GGGAATTTGGCGACGTAATTC
ERCC1 reverse	GCGGAGGCTGAGGAACGA

### Western blot analysis

Cells were lysed in RIPA buffer (50 mM Tris-Cl at pH 7.5, 150 mM NaCl, 1% NP-40, 0.5% sodium deoxycholate, 1 mM EDTA, 1 μg/mL leupeptin, 1 μg/mL aprotinin, 0.2 mM PMSF) and proteins (20–40 μg) were separated on 8–10% SDS/PAGE gel and then transferred onto PVDF membranes (Millipore, Billerica, MA). After blocking procedure, membranes were incubated with primary antibodies (1:1000), HRP-conjugated secondary antibodies (1:5000), and visualized in Imager (Bio-Rad) using ECL system (Thermo Fisher Scientific, Rochester, NY). Antibodies of GAPDH, Bcl-2, Bcl-xL, Bax, and p53 were purchased from Santa Cruz Biotechnology (Santa Cruz, CA), and antibodies of Mcl-1, p-Akt, p-Erk, p-Stat3, and p-MAPK were purchased from Cell Signaling (Danvers, MA). Antibodies of ATM, CHEK1, TP73, and ERCC1 were obtained from Bethyl Laboratories (Montgomery, TX).

### *In vivo* xenograft studies

The A549sc and A549IL-6si cells (1 × 10^6^/site) were subcutaneously injected into flanks of 8 week old female nude mice (NCI) (10 mice per group, total 20 mice). Tumor development and volumes were measured twice a week. When tumor volumes reached 400 mm^3^, cisplatin (3 mg/kg) were i.p. injected two times per week and tumor growth was monitored. At the end of treatment, mice were sacrificed and tumor tissues were processed for staining.

### Histology and immunohistochemistry

Tissues obtained were fixed in 10% (v/v) formaldehyde in PBS, embedded in paraffin, and cut into 5-μm sections. Tumor tissue sections were deparaffinized in xylene solution, rehydrated, and immunostaining was performed using the IHC kit (Santa Cruz, Santa Cruz, CA). Antibodies of IL-6 (Sigma, St. Louis, MO), Ki67 (Abcam, Cambridge, MA), Bcl-2 (Santa Cruz, Santa Cruz, CA), Mcl-1 (Cell Signaling, Danvers, MA), and ATM and CHK1 (Bethyl Laboratories, Montgomery, TX) (all antibodies at 1:250 dilution) were applied in staining. For Ki67 staining, the antigen retrieval process was performed in 10 mM Citric buffer, pH 6.0 for 20 minutes using a cooker prior to staining. After staining, tissues were counterstained by Hematoxylin.

### Statistics

The data values were presented as the mean ± SEM. Differences in mean values between two groups were analyzed by two-tailed Student's *t* test. *p* ≤ 0.05 was considered statistically significant.
